# Influencing factors and reduction of domestic solid waste at university dormitory in Shanghai, China

**DOI:** 10.1038/s41598-021-04582-0

**Published:** 2022-01-12

**Authors:** Yuhan Pan, Mengyang Li, Hongwei Guo, Yuanyuan Li, Ji Han

**Affiliations:** 1 Shanghai Key Laboratory for Urban Ecological Processes and Eco-Restoration, School of Ecological and Environmental Sciences, East China Normal University, Dongchuan Rd. 500, Shanghai, 200241 China; 2 Logistics Support Department, East China Normal University, Dongchuan Rd. 500, Shanghai, 200241 China; 3 Institute of Eco-Chongming, 3663 N. Zhongshan Rd., Shanghai, 200062 China

**Keywords:** Energy conservation, Energy management, Sustainability

## Abstract

Increasing domestic solid waste (DSW) is becoming one of the most serious challenges for city and regional environment. As an epitome of the society, the investigation on the influencing factors and reduction of DSW of university students can not only provide policy suggestions for the waste management in the university campus, but also can achieve demonstration effect to other communities due to its high social status and wide impacts. This research combined direct weighing, questionnaire surveys, and regression analysis to quantify the influencing factors of DSW at East China Normal University’s dormitory in Shanghai. Direct weighting and questionnaire survey were conducted in 112 randomly selected dormitory rooms. Totally 523 valid questionnaires were collected. It is found that the average waste generation was 0.275 kg/day/cap, in which residual waste accounted for 64% of total, followed by household food waste (29%), and recyclable waste (7%). Regressions based on ordinary least square method suggested that students’ attitude towards waste played the most important role in affecting the waste reduction with its elasticity − 0.195. Lower educational level and better financial condition would lead to more waste generation, whose elasticity was 0.148 and 0.098 respectively. The influences of gender and major varied from waste types. Policies implications for university administration departments for sustainable waste and resource management include developing personalized and humanized waste management policies, enhancing environmental awareness through diverse educational activities, and expanding the publicity role of campus cultural activities on waste reduction.

## Introduction

Booming economy, population growth, and consumption pattern change have greatly urged the production of domestic solid waste (DSW), especially in developing countries^1–3^. China has reported an 890% soaring of DSW from 1979 to 2018^4^ and a continued growth rate of 8–10% per annum in the future^5^. It leads to the dilemma that nearly 2/3 of China’s cities have become “garbage-besieged cities”^6^. At the same time, due to the often-happened mixture of hazardous materials, such as electronic waste and batteries with DSW, it is easy to cause pollution and health risks. Thus, if handled improperly, DSW will cause serious harm to the environment and human society, which threatens the long-term sustainable development of cities^7^.

As an epitome of the society, university has brought about increasingly negative environmental problems along with the enrollment expansion. In 2019, there were 2879 colleges and universities in China, with the total number of students reached 30 million. The average daily output of DSW was about 0.5 kg^8^, which means the total amount of university waste generation was about 15 thousand tons per day and 5.5 million tons per year. However, very few studies have taken universities as the research subject to explore the influencing factors and reduction of DSW. Meanwhile, some studies have found that the majority of people who have positive attitudes towards environmental behavior are under the age of 24^9^. University students are just the group with awareness and willingness to take the action of environmental protection. Therefore, the investigation on the influencing factors and reduction of DSW of university students can not only provide policy suggestions for the waste management in the university campus, but also can achieve demonstration effect to other communities due to the high social status and wide impact of universities, which is expected to contribute to the waste management and sustainable development of cities.

Differing from the existing literature, this study highlighted an investigation of DSW issue at university level of China. Through extending the theoretical framework of planned behavior in the context of waste management, the effect of attitude towards the behavior, perceived behavioral control, and subjective norms, together with personal attributes such as gender, major, grade, and living expenses are investigated quantitatively. The objectives are (1) to investigate the influencing factors of DSW at university in China, (2) to explored the potential and policy implications for waste reduction. Our results can help to understand the university students’ attitude and behavior in dealing with DSW, and provide a basis for policy makers in designing regulations for reducing waste and better managing resources.

## Literature review

Studies on the influencing factors and reduction of DSW is not only one of the social concerns, but also the hot spot of many research disciplines such as ecology, environment, management, and sociology, etc. Scholars have conducted numerous studies on the topic at various scales in the world (as summarized in Table 1). Generally, it is found that socio-demographic, economic, urban development, human behavior and etc. are the dominant factors affecting the production of DSW.

**Table 1:** Recent studies on the influencing factors of DSW.

Scale	Authors	Year	Study area	Influencing factors
Country	Zhao et al.	2016	China	population, urban built-up area, GDP, per capita expenditure on consumption, per capita disposable income
	Cheng et al.	2020	China	population size, economic, urbanization level, industrial structure
	Daskalopou-los et al.	1998	Europe and USA	GDP, population
	Javier et al.	2015	Europe	tourism quantity, quality and specialization
	Namlis et al.	2019	Europe	GDP, Human Development Index, unemployment rate, carbon dioxide emission
	Saniye et al.	2012	Turkey	unemployment rate, proportion of asphalt pavement
City/region	Rémi et al.	2018	Vaud, Switzerland	per capita income, urbanization level, policy mechanism
	Vieira et al.	2018	Sao Paulo, Brazil	per capita income
	Patel et al.	2013	Gujarat, India	population size, per capita income, local maximum temperature
	Abdoli et al.	2012	Mashhad, Iran	economic prospects, residential spending, infrastructure investment
	Oribe-Garcia et al.	2015	Biscay, Spain	urban morphology, tourism activity, level of education, economic situation
	Lebersorger et al.	2011	Styria, Austria	population size, urbanization level, per capita disposable income
Community/household	Xiao et al.	2015	191 household in Xiamen, China	family structure and lifestyle
	Afon et al.	2016	648 households in OYO, Nigeria	income, household size, social status, occupation, education level
	Monavari et al.	2012	400 households in Ahvaz, Iran	household size, occupation, age, number of rooms, education level
	Mohamad et al.	2020	300 households in Homs City, Syria	income, household size, age, education level of head of the family
	Liu et al.	2017	Foshan, Guangdong, China	subjective norms, behavioral attitudes to waste
	Tam et al.	2008	Hongkong	political measures
	Yang et al.	2020	/	reactive action
	Botetzagias et al.	2015	Greek	perceived behavioral control

At the national scale, Zhao et al.^10^ observed a significant and positive correlation between China’s DSW generation and factors such as urban population, GDP, urban built-up area and per capita disposable income. Cheng et al.^11^ conducted ordinary least squares analysis, and found that with the increase of population size, per capita GDP, urbanization level and the proportion of tertiary industry in GDP, the generation of DSW in China also increased correspondingly. Across United States and some countries of Europe, a strong correlation between domestic waste production and GDP and population was found by Daskalopoulos et al.^12^. Javier et al.^13^ evaluated the impact of tourism volume, tourism quality and tourism specialization on DSW based on the data of 32 EU member countries from 1997 to 2010, and confirmed the nonlinear impact of tourism arrivals, per capita tourism expenditure and tourism specialization on DSW. Namlis et al.^14^ studied the effects of GDP, human development index, unemployment rate and carbon dioxide emissions on the production rate of solid waste in 10 EU countries since the economic crisis, and found a positive correlation between GDP, carbon dioxide emissions and the production rate of DSW. Saniye et al.^15^ also found that urban solid waste generation in Turkey was significantly correlated with unemployment rate and the proportion of asphalt pavement.

At the city scale, Remi et al.^16^ found that a direct policy mechanism for example, the waste bag tax was significantly correlated with a decrease in waste generation in Vaud, Switzerland. Vieira et al.^17^ explored the impacts of socioeconomic factors on DSW generation in Greater Sao Paulo, and found inequality may explain the differences of waste generation between areas with similar incomes. Patel et al.^18^ considered both socio-economic and physiographic factors in explaining the driving factors of DSW in Gujarat of India, and found that population size, economic level and geographical location (latitude and longitude) were significant influencing factors. Oribe-Garcia et al.^19^ explored the social and economic characteristics related to DSW production in Biscay by means of factor models. They identified urban morphology, tourism activity, level of education and economic situation as the most influential factors in waste generation. Abdoli et al.^20^ evaluated the influence of social, economic and environmental factors on DSW output with multiple regression model, and concluded that economic prospects, residential spending, infrastructure investment were the important influencing factors. Lebersorger et al.^21^ investigated 116 factors influencing the generation of waste in Styria and found that household size, municipal tax revenue per capita and the percentage of buildings with solid fuel heating systems were the most significant factors.

Besides, a number of studies have been carried out at community and household scales. Xiao et al.^22^ selected 191 households in rapidly urbanized areas in Xiamen of China as research samples, and observed that household structure and lifestyle led to significant differences in waste generation in work-unit, transitional, and commercial communities. Afon et al.^23^ employed OLS regression analysis and found that family size, social status, occupation, education and season significantly affected waste production in Oyo. Based on a sample of 400 families in Iran, it was confirmed that higher employment rate, education level and age were, the less DSW were generated^24^. Abu Qdais et al.^25^ conducted a survey on the basis of linear regression, and found that there was a significant positive correlation between waste output and income level. The higher the income of residents, the more garbage discharged. The waste generation of high-income family was about 35% higher than the average. Taking residents in Homs, Syria as the research object, DSW was positively correlated with monthly household income, family size and age of the head of the household, and negatively correlated with the education level of the head of the household^26^.

At the scale of residents, there are also many studies on the influencing factors of the behavior of reducing and recycling waste. Liu et al.^27^ analyzed the influencing factors in construction worker's willingness to reduce construction waste, and found subjective norms and behavioral attitudes towards construction waste have a direct impact on behavioral intention in construction reducing. Tam^28^ took Hong Kong as an example and found that “waste emission reduction system” achieved good implementation effect. Yang et al. developed a simulation model through a system dynamics approach to explore the causation of waste reduction behaviors and found that reactive actions was the most significant^29^. Botetzagias et al.^30^ found that perceived behavioral control is the most important predictor of the intention to recycle and demographic characteristics were found to be statistically non-significant.

Researches on influencing factors of waste generation are abundant and in-depth, and cover different scales, such as country, city, and community. Nevertheless, few studies were conducted at university level. University students are a unique group of young people shouldering the responsibility for sustainability in China’s future^31^. Moreover, universities can be regarded as a community composed by young and highly educated people from all over the China, having the moral responsibility to move towards more sustainable modes of life^32^. The implications of reducing waste in universities can also be applied to other communities^33^.

Shanghai, as a first-tier city in China, has a quite high education level, thus drawing numerous excellent students from all over China. Accordingly, universities in Shanghai serve as representative samples for understanding the waste behaviors of university students in China.

## Methods and data

### Theoretical framework

**Figure 1:**
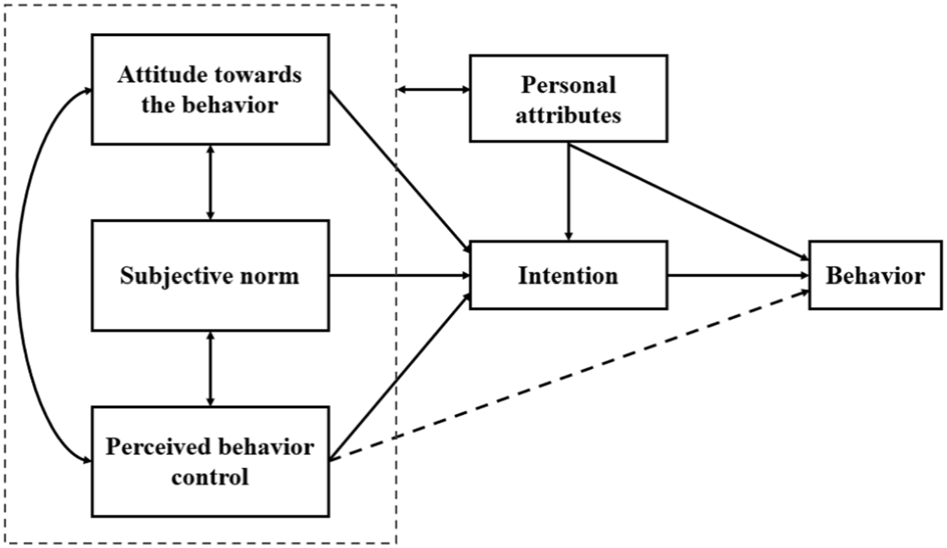
Theoretical framework of the extended theory of planned behavior. *Note* Attitude towards the behavior refers to people’s positive or negative feelings towards the behavior in question. Perceived behavioral control indicates people’s perception of ease or difficulty in performing certain behaviors. Subjective norms reflect the individual’s perception that people who is important to the individual should perform the behavior. Personal attributes cover gender, major, grade and financial condition. etc.

The theory of planned behavior (TPB), which claims that behavior intention predicts individual behavior, has been widely employed in analyzing human behavior of interest^34,35^ especially for students and green consumers^36,37^. According to TPB, behavior intentions can be further determined by three factors (shown in Fig. 1), i.e. attitude towards the behavior (ATT), perceived behavioral control (PBC), and subjective norms (SN)^38,39^. ATT refers to people’s positive or negative feelings towards the behavior in question. PBC indicates people’s perception of ease or difficulty in performing certain behaviors. SN reflects the individual’s perception that people who is important to the individual should perform the behavior.

In general, more positive attitudes and subjective norms, associated with perceived behavioral control, can enhance an individual’s intention to perform a specific behavior^40^. Tonglet et al.^34^ argued that recycling attitude influence waste recycling significantly, Dwivedy and Mittal proved that the convenience was the most significant influencing factors on resident behavior^41^. In addition, research shows that subjective norms have an indirect effect on behavioral intentions through behavioral attitudes and perceived behavioral control^42^. In addition, personal attributes such as gender, major, education level and financial condition are also influencing factors of individual’s behavior, thus we extended the theory and selected attitude towards the behavior, perceived behavioral control, subjective norms and personal attributes as measures thereof.

### Study area and data collection

In this study, East China Normal University (ECNU), which is one of the most prestigious universities in China with a total registered student number over 34 thousand in 2021, is selected as a case study university. The reasons for choosing this university lie in two aspects. First, it is the first normal university established after the founding of China in 1949, and was chosen as one of the Double First-class Universities by the central government in 2017. It is famous for fostering teachers from kindergarten to high school at the beginning, and now has rapidly developed into a comprehensive research university. Second, the Shanghai city where the university is located is another important reason as Shanghai is one of the biggest cities and also the first city in China bringing waste classification and recycle into force by law. In 2019, Shanghai municipal government promulgated the Regulations on the Administration of Municipal Domestic Waste to the whole society in order to tackle the problems posed by DSW. It minimizes waste from the source and enhances the recycling efficiency. It is also an important way to realize the recycling, reduction and harmlessness of DSW. Thus, investigating the influencing factors and reduction of DSW at a top-class university in a pioneer city implementing waste classification and management will help to have an in-depth understanding of the people’s environmental behavior so as to develop proper policies toward sustainable waste management.

In this study, we basically collected two kinds of data for analysis. One is the quantity and composition of DSW. The other is the information of students that will influence the generation and reduction of DSW.

Regarding the former, we adopted the direct weighing of waste generation in each randomly selected dormitory room. Following the instruction of Shanghai’s waste classification standard, three major types of waste were investigated, i.e. household food waste, residual waste, and recyclable waste. Due to the very limited amount of hazardous waste generation of student dormitory, this type was not considered in our analysis. Within the campus, the waste dumping sites are open in three time slots from 7 a.m. to 9 a.m., 11:30 a.m. to 13:30 p.m., and 17:00 p.m. to 19:00 p.m. All the students are required to dump their DSW within the fixed time. With the approval and help of the logistics support department of the university, we are permitted to enter the dormitory and conducted waste weighting in those randomly selected student rooms during the waste dumping time slot. The selection of the dormitory room was based on the criteria that the survey samples should cover the diversity of majors, different grade of both undergraduate and graduate, gender of the students. The field survey was carried out from November, 2018 to June, 2019.

Regarding the influencing factors of DSW, we designed a questionnaire based on the theoretical framework shown in Fig. 1 and previous studies^43,44^. The questionnaire survey was approved by the University Committee on Human Research Protection (UCHRP) of ECNU, and all methods were carried out in accordance with the regulations of UCHRP. Informed consent was obtained from each interviewed student. As shown in the Appendix A, the questions included three aspects, personal attributes, personal choice factors, and attitudes towards waste generation. First, the personal attributes are composed by the following objective factors, gender (male/female), grade (undergraduate/master/doctoral), major (in accordance with the 12 main disciplines released by the Chinese government), living expenditure per month (< 500 CNY; 500–1000 CNY; 1000–1500 CNY; 1500–2000 CNY; > 2000 CNY).

Second, the personal choice factors are intended to judge the students’ subjective choices. It includes the time they produce waste, the reasons why the waste in the dormitory increase, behaviors that may reduce the waste in the dormitory, and the preferred ways that they are willing to participate in waste recycling and resource utilization. The detailed questions about personal choice factors can be found in the Appendix A.

Third, the students’ attitudes towards waste generation are measured using a Likert 5-step agreement level (in which 1 represented full disagreement and 5 represented full agreement). The total 6 statements are as follows: (1) The regulations of waste classification and reduction may restrict my waste generation; (2) Waste classification in dormitory room is important; (3) It will waste my time and effort to do the waste classification and reduction in my dormitory; (4) Waste source classification can reduce the amount of DSW and environmental pollution; (5) My roommates can correctly do the waste sorting in the dormitory; (6) The cooperation between my roommates and I may affect my participation in waste classification, reduction and resource utilization.

### Regression analysis

To quantitatively show the influences of factors on DSW, we set up a regression model using an ordinary least square (OLS) method because the dependent variable (*Waste*), measured by the amount of waste, was a continuous variable. The details are illustrated in Eq. (1)

1 $${Waste}_{i}=\alpha +\eta {ATT}_{i}+\theta {PBC}_{i}+\xi {SN}_{i}+\rho {PA}_{i}+{\varepsilon }_{i}$$

where the subscript *i* represents the sample of each surveyed student; *α* is a constant; *η, θ, ξ, ρ* are the coefficients; *ε* is the error term that we assumed obeyed normal distribution. To predict the waste generation behavior of students, we chose three questions in the questionnaire that are most relevant to ATT, PBC and SN. ATT was measured by the score of student’s responses to the statement “Waste source classification can reduce the amount of DSW and environmental pollution”. PBC is measured by the score of response to the statement that “The regulations of waste classification and reduction may restrict my waste generation”; The higher score the better attitude towards reducing waste generation. SN is measured by the score of response to the statement that “The cooperation between my roommates and I may affect my participation in waste classification, reduction and resource utilization”. The higher score the more easily influenced of the students by their surrounding people. In addition, some studies found that demographic factors are closely related with green behavior in China^45,46^. Thus, we also considered personal attributes (PA) as an influencing factor of waste generation. Gender (Gender), major (Major), grade (Grade) and living expenses (Expense) are selected as the explanatory variables. Table 2 gives the definition of all variables.

**Table 2:** Definitions of all the variables in regression analysis.

Variable	Definition
Waste (waste)	The weighed waste of each observation (kg)
Attitude towards behavior (ATT)	Discrete and ordered variable, low to high (1–5)
Perceived behavioral control (PBC)	Discrete and ordered variable, low to high (1–5)
Subjective norms (SN)	Discrete and ordered variable, low to high (1–5)
**Personal attributes (PA)**	
Gender (gender)	Male, *Gender* = 0; Female, *Gender* = 1
Major (major)	Natural science, *Major* = 0; Social science, *Major* = 1
Grade level (grade)	Discrete and ordered variable, 1, 2, 3 stands for undergraduate, master, and doctoral respectively
Living expense (expense)	Discrete and ordered variable, low to high (1–5)

We employed six regressions based on Eq. (1). The parameters were estimated by using the ordinary least squares (OLS) method with robust standard errors, which minimize the sum of the squares of the differences between dependent variable and independent variables. This approach provides the “Best Linear Unbiased Estimator” when the independent variables are exogenous and the errors are homoscedastic and serially uncorrelated^47^. We firstly conducted the regression based on the TPB framework, which included only ATT, PBC, and SN. Then, we further controlled the impact of personal attributes in the second regression. The goodness-of-fit for each regression was measured by the *R*^2^, through which we could further detect the extent of changes in explanatory power for the model that includes PA in addition to TPB framework. Furthermore, we replaced the dependent variable (Waste) by two major waste types, i.e. household food waste (Waste_h), and residual waste (Waste_r).

## Results

### Descriptive analysis of influencing factors

Totally, 112 dormitory rooms were investigated and 523 valid questionnaires were collected. The personal attributes of the surveyed samples are given in Table 3.

**Table 3:** Personal attributes of the surveyed samples.

Attribute	Percentage (%)
**Gender**	
Male	35.00
Female	65.00
**Grade**	
Undergraduate	70.21
Master	16.25
Doctoral	13.54
**Major**	
Natural science	43.54
Social science	56.46
**Living expenses per month**	
< 500 CNY	0.42
500–1000 CNY	6.88
1000–1500 CNY	29.58
1500–2000 CNY	27.71
> 2000 CNY	35.42

**Figure 2:**
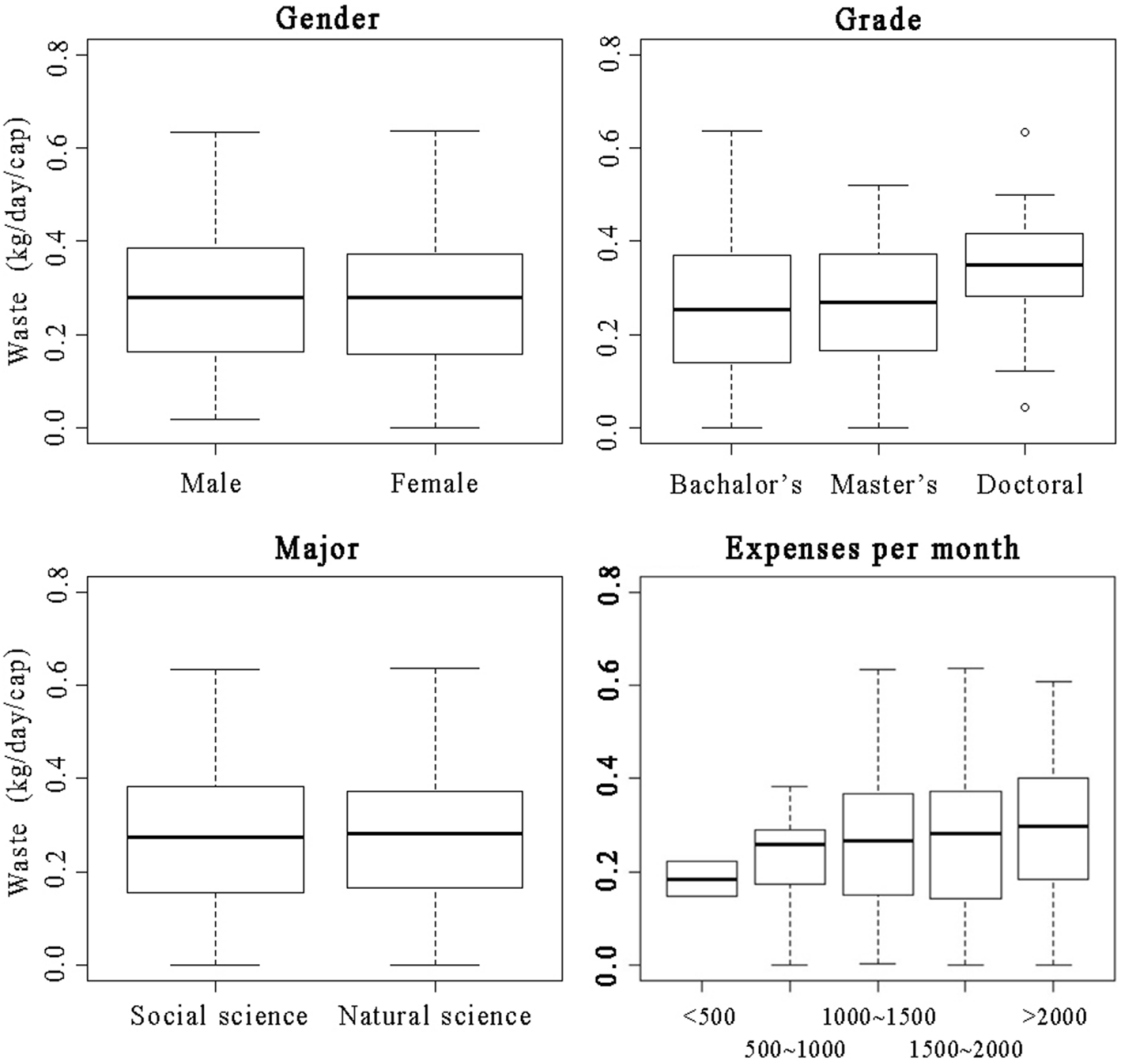
Descriptive results of personal attributes effect on waste generation. *Note* the bold and black line indicates the average waste generation of students of different gender, educational level, major and financial condition.

According to the survey, the average waste generation of one university student was 0.275 kg/day, among which residual waste accounted for 64% of total, followed by household food waste (29%), and recyclable waste (7%). Generally, as shown in Fig. 2, the difference of waste generation was not significant by gender and major, but noticeable by grade and living expenses per month. Doctoral student generated more waste (0.34 kg/day/cap), which is 26% higher than that of master student (0.27 kg), 31% higher than that of undergraduate (0.26 kg/day/cap). With the monthly living expense increases, the waste generation also showed an upward trend. Students with expenses less than 500 CNY produced much lower waste than others, which was only 0.19 kg/day/cap, and 31% lower than the average. Students with expenses larger than 2000 CNY generated relatively higher waste, which was about 0.29 kg/day/cap, and 5% higher than the average.

### Regression results of influencing factors

As illustrated in Table 4, the regression models incorporating the impact of three aspects of TPB framework with personal attributes (PA) had a relatively better performance than those only based on TPB theory. The relatively higher *R*^2^ indicates a better ability to predict the waste generation behavior of university students. In regression model 2, ATT and PBC had a negative impact on waste generation at the 1% and 5% significance level, which means that the more a student agree with the statements “Waste classification can reduce the amount of DSW and environmental pollution” and “The regulations on waste classification and reduction may restrict my waste generation”, the less waste would be generated. With regard to personal attributes, the regression results further verified the findings observed in Fig. 2. Both the gender and major had insignificant impacts on waste generation behavior of students. Grade and expense had a significant and positive impact. In sum, the waste generation behavior of university students was most relevant to the attitude towards waste, and was also significantly influenced by grade level and financial condition.

**Table 4:** Regression results of influencing factors of DSW.

Variables	Total waste		Household food waste		Residual waste	
	1	2	3	4	1	2
ATT	− 0.209*** (− 4.593)	− 0.195*** (− 4.271)	− 0.159*** (− 3.451)	− 0.16*** (− 3.551)	− 0.06 (− 1.288)	− 0.046 (− 0.997)
PBC	− 0.096** (− 2.139)	− 0.089** (− 1.991)	− 0.035 (− 0.775)	− 0.044 (− 1.012)	− 0.102** (− 2.249)	− 0.088 (− 1.954)
SN	0.007 (0.150)	− 0.001 (− 0.032)	0.058 (1.246)	0.053 (1.197)	− 0.044 (− 0.948)	− 0.057 (− 1.252)
Gender		− 0.005 (− 0.103)		− 0.116** (− 2.472)		0.017** (0.355)
Major		0.033 (0.696)		0.205*** (4.330)		− 0.166*** (− 3.433)
Grade		0.148*** (3.236)		0.14*** (3.112)		0.107** (2.334)
Expense		0.098** (2.202)		0.071 (1.624)		0.012 (0.272)
*R*^2^	0.048	0.07	0.02	0.094	0.012	0.053
F-statistic	9.107	6.129	4.336	8.080	2.937	4.796

Value is the standardized beta coefficients. *t* statistics in parentheses.

***, ** and * stands for the significance at 1%, 5% and 10% levels respectively. 1–6 refers to different regression models, in which the odd number means the regression does not consider personal attributes’ effect, while the even number means the regression considers.

Differing from the total waste, the influencing factors varied from waste types. As shown by the results of model 3–4, gender had a significant and positive effect on residual waste, indicating that female students produced more residual waste than male students. This may be attributed to the preference of online shopping, which will generate a large amount of packaging materials as residual waste. Students from natural sciences produced more residual waste than those from social sciences. However, the impacts of gender and major showed a completely opposite effect in household food waste generation. As shown in the results of model 5–6, male students and those studying social sciences produced more household food waste than female and natural sciences. In terms of residual waste, major, grade and PBC are the relatively more important factors driving the waste generation. Major, ATT and grade played a dominant role in affecting the generation of household food waste.

### Effects of personal choice factors

**Figure 3:**
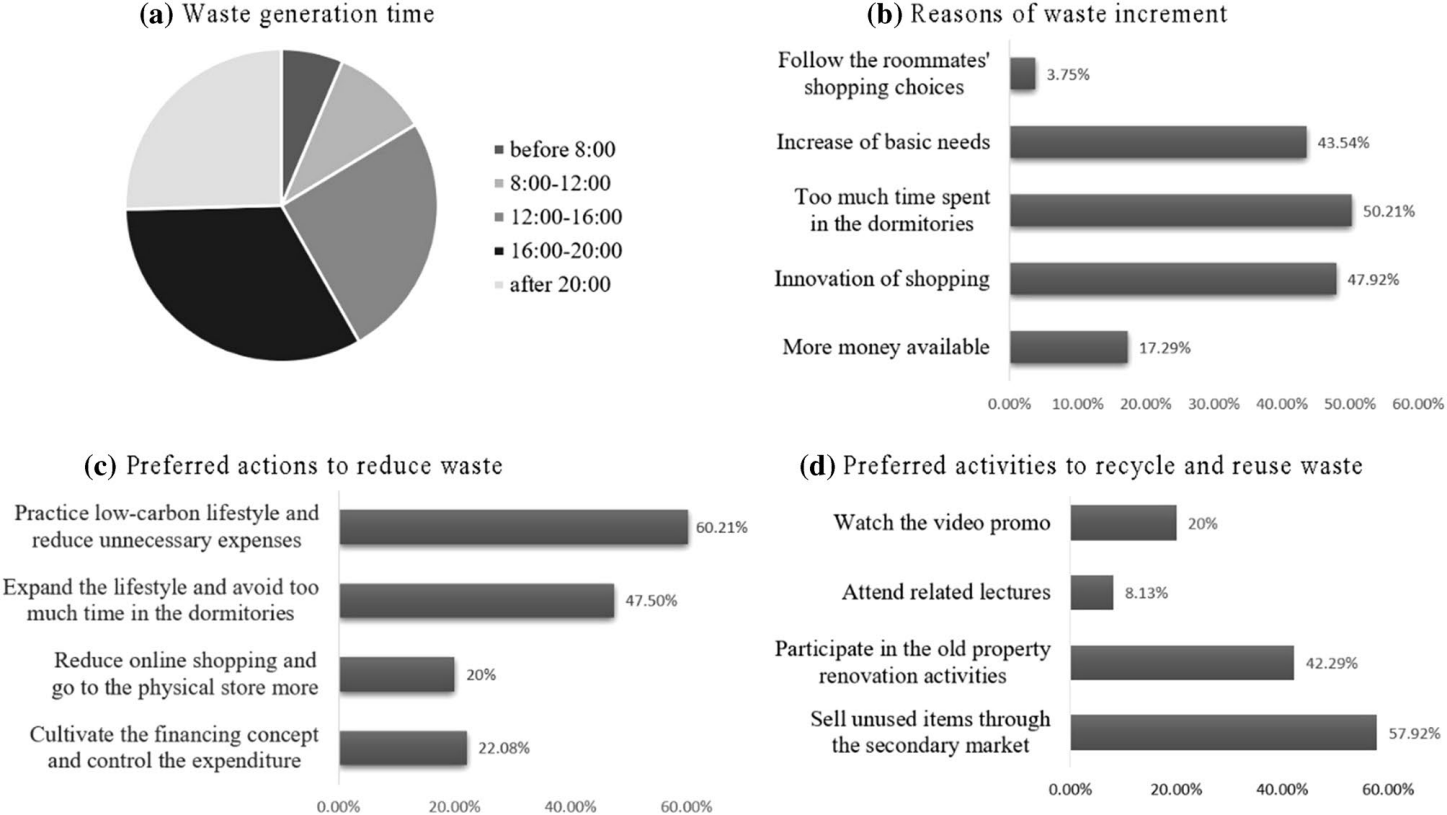
Results of personal choice factors. (**a**) Proportion of waste generation time period of respondents; (**b**) reasons of students generated more waste; (**c**) actions students preferred to take to reduce waste; (**d**) activities students preferred to take part in to recycle and reuse waste.

In addition to analyzing the factors of TPB framework and personal attributes, we also assessed the impact of personal choice factors on DSW. As shown in Fig. 3, it includes the time generating waste in the dormitory, the causes of the waste increment, the actions may take to avoid more waste generation, and the waste recycling and reduction activities.

According to the survey, one-third of the students produced waste between 4 p.m. and 8 p.m., while about a quarter produced waste during 12 p.m.–4 p.m., and after 8 p.m., respectively. It indicated that the intensive time slots that students produced DSW are in the afternoon and evening.

More than half of the respondents thought too much time spent in the dormitory was the most essential reason for increasing waste generation. 48% and 44% of the students thought that the innovation of shopping ways such as online shopping and the increase of consumption needs will also lead to the increase of waste generation. This phenomenon is widely observed in the rapid developing countries and regions, as the improvement of the economic level and living standard will stimulate the consumption needs. At the same time, it’s worth noting that following the roommate’s shopping choice did not affect the waste generation behavior of students in the dormitories. Only less than 5% of the students picked that option, indicating that the behavior of surrounding people will not cause influence on students in the dormitory. It is also consistent with the regression result that the subjective norm (SN) was not statistically significant.

Regarding the question what kind of activities the students may take to avoid producing more waste, 60% of respondents were willing to practice a low-carbon lifestyle and cut back on unnecessary spending in their daily life. Nearly half of the students (48%) would like to vitalize their lifestyle and spend less time in the dormitory. Cultivating the awareness of financial management, controlling expenditure and reducing the frequency of online shopping were also chosen by about 20% of students to reduce the amount of waste production, respectively.

In addition to reduce DSW at the source, recycle and reuse of waste were also important for achieving sustainable waste management. 58% of the respondents were willing to sell useless and unused items through the flea market, as they thought it can not only reduce the waste but can also gain some pocket money. 42% of students would participate in the recycling and creation activities under the guidance of craftsmen to turn the unused items into handicrafts. Differing from our expectation, though the Shanghai city and the university have attached great importance in propagating waste classification and reduction, less than 20% of students were willing to participate in those educational activities such as lectures and videos related to DSW reduction.

In addition, we also leave an open question in the questionnaire for students about the difficulty in waste classification and reduction. The answers can be summarized into the following aspects. Missing the waste dumping time slots thus unable to throw the waste; unclear about the regulations on waste classification and reduction, and a little far away from the waste dump sites, etc.

## Discussions and policy suggestions

### Policy discussions and suggestions

With the development of economy and the improvement of people's living standards, the young generation of China view problems with a more open vision and pay more attention to the environment problems. ECNU, as a first-class comprehensive university in China, whose students shared lot in common with those from foreign universities, such as the increased preference in online shopping, more willingness to participate in a low-carbon life style, and recycling and reusing the unused items. Therefore, our study can not only explore the influencing factors and reduction measures of waste generation of Chinese university students, but also provides experience for international comparative studies and suggestions for waste management in the whole world.

Based on the descriptive and regression analyses of influencing factors, several policy suggestions facing the university student’s waste management can be derived.

Among the personal attributes, financial condition played an important role in affecting student’s waste generation. In recent decades, China’s economic growth has gained world attention, and so has the living expenses of students. The per capita income in China has increased remarkably from 19,109 to 43,834 CNY in the last decade^48,49^. The improvement of living expense increased the purchasing power of students, leading to a significant increase of waste^50^. It is an evitable trend as many developed countries have the same experiences^51,52^. Obviously, it is expected to reduce the waste through fostering an environmentally friendly consumption behavior and providing effective services rather than restricting the living expense.

Interestingly, doctoral students were found to generate much more waste than master and undergraduate students. It may be attributed by more time spent in the dormitories, as they have less lessons to take and have more free time to control by themselves. At the same time, to some extent, grade can reflect the educational level of the respondents. Similar findings were also observed in other case studies. For example, Li et al.^53^ found that at the community level the average waste output per day showed an upward trend with the improvement of the educational level. Students are a special population group who are easily transform ideas into actions if they had a clear understanding of the environmental and economic costs caused by DSW. At the same time, the high degree of recognition of PBC-related issues reflected that the current waste classification policy did restrict students' waste production. Some studies in China also found that authority’s participation is very important for improving people’s perception of a program^54^. Thus, in order to turn environmental awareness into behavioral change more effectively, society and university should work together to better educate students especially the high-grade students on the environmental impacts of waste, and encourage them to participate in waste reduction related activities and improve their public awareness of responsibility of waste classification and reduction.

Since the gender has been found having influences on different waste types, for the waste management department of university, attentions should be paid to female dormitory regarding the residual waste, while male dormitory for household food waste. In the existing literature, there is a debate whether the major of students will affect the students’ awareness to protect the environment. Some believed that there was no difference between social sciences and natural sciences, as social connection, experiences, socio-economic conditions are the key factors influencing environmental behavior^55^. Some observed significant difference in the perception of negative effects of waste and the willingness to reduce waste among students with different major. A case study of National Chung Hsing University in Taiwan revealed that students majored in natural sciences had a better performance in waste reduction as more courses related to environment are offered^56^. In our study, the influence of major also differed in waste type. More researches need to be carried out in the future.

As the survey suggests, the mismatch between the waste generation time and the opening hours of dumping site, and the far distance from dormitory to the dumping site are pointed by the students as the two most difficulties during waste classification and reduction. The fixed opening time of dumping site may constrain the convenience of students to throw garbage. As possible countermeasures, on one hand encouraging students to develop good habits of waste classification and dumping within the regular time is an important and long-term effort of university. On the other hand, setting up some specific dumping sites opening for those students who do have difficulties in throwing waste during the regular time, such as those have to leave or return the dormitory very early or late due to the research needs. Though it may increase the management cost of university, it is also in line with the trend of constructing a student-oriented management of most universities. Regarding the far distance problem, many countries have formulated relevant norms and standards of site choices of waste dumping site. The standards on waste recycling developed by the Environmental Protection Association of the United States have explicitly stated the space requirements and size of the waste collection containers^57^. In our case, it is possible for the university to integrate the data of waste generation within the campus with some geographic information system (GIS) models so that the spatial patterns of waste can be monitored and predicted. On the model basis, the best location of dumping sites could be optimized and the far distance problem could be solved.

Since the attitude towards the behavior has a significant impact on waste reduction, to cultivate the student’s environmental awareness is vital for waste management. However, the questionnaire result indicated only less than 20% of students were willing to participate in those educational activities related to DSW reduction. It is big challenges for university to enhance student’s awareness to reduce, reuse, and recycle of DSW. The experience of the City College of San Francisco (CCSF) in the United States is worth learning from. CCSF offers courses on the theme of “Campus Waste Reduction and Resource Recycling” in the curriculum of art and advertising majors^58^. Students can not only use the knowledge learned from the class to effectively convey the awareness of environmental protection to other students, but also provide a guarantee for the professional and long-term development of campus waste reduction and recycling propaganda.

In ECNU, the university has already opened several public courses teaching environmental and ecological theories. In addition to opening more public and fundamental courses related to waste and resource management, some elective courses could be designed combining theories with practices so as to attract more students. Camosun College in British Columbia established a food waste composting course in 2003, using campus food waste for composting^59^. The coordinated project is conducive to the long-term and stable operation of campus food waste management. Students can gain lots of benefits such as knowledge learning, and technique practicing. Besides, some special lectures and waste reduction related knowledge competition could be held regularly on the basis of some big events, such as the World Environment Day, and World Earth Day. Through inviting professionals and well-known scholars to educate students the research hotspots and cutting-edge progress in waste disposal and management will provide diverse forms of educational activities for students to participate in.

Since our results suggested that more than half of students were willing to recycle idle goods and waste, university could organize some culture activities and expand their functions from vitalizing campus life to the publicity of environmental projection. The activities may include the flea market, culture and creative products design contest, DIY dormitory waste-oriented product competition, and etc.

In ECNU, the flea market does exist. However, the market scale and form could be further expanded, and could be held regularly both online and offline. Students in the survey expressed strong willingness and interests in recycling and exchanging idle goods through the market. It not only prolongs the life time of goods thus reduces waste generation from the source, but also provides a social exchange platform. In the internet era, waste reduction in universities should give full play to the advantages of the network and expand the market to online form. WeChat, which is one of the most popular social networking apps loved by young generation, could be used to build an online second-hand goods trading and recycling platform under the guidance of the university supervision.

To vitalize the student’s campus life, the logistics support department of ECNU has organized some culture and creative events within the dormitory. For example, they invited some craftsmen to teach students how to utilize the waste or unused items as materials and design them into handicrafts, such as pet clothes made from old clothes, flowerpot made from recycled plastics. It was very popular and welcomed by the students as they had fun and a sense of fulfillment. More importantly, they perceived the value of waste and enhanced their environmental awareness. Such activities could be organized regularly and introduced to student society to expand their impacts.

### Limitations and future works

Firstly, due to the difficulties in data collection, there were only 112 dormitories, 523 selected samples in the study, in which undergraduates accounted for a large proportion. It may have influence on the waste generation output of students with different education levels. Further study is thus needed to pay attention to the proportion of survey samples.

Secondly, East China Normal University, as a first-class university in China, has a wide range of students from different China regions and even from foreign countries. Students from different social backgrounds differed in many aspects, such as environmental awareness and knowledge structure. However, we didn’t consider the geographic differences and impacts of students’ origins in this research. In future study, it is necessary to consider the place of origin as an influencing factor to explore whether there are differences in individual behaviors of students under the socioeconomic background of different hometowns.

Finally, due to the impact of COVID-19, our questionnaire survey duration was limited, and covered from December 2018 to June 2019. Longer duration covering the whole year is expected as it may reflect the seasonal impact on waste generation and reduction. In the post-epidemic era, the importance of online survey becomes more prominent. The advantages of the internet should be fully exploited, such as online questionnaire survey. Also, the survey ought to be carried out in different periods considering the impact of seasons and semesters to make the results more comprehensive.

## Conclusions

Realizing the reduce, reuse and recycle of domestic solid waste is a big challenge and also a sustainable development goal for cities and regions in the world. Revealing the influencing factors of DSW is the basis for understanding people’s behavior and attitude toward waste issue, so that proper policies and regulations could be developed. Differing from the existing literature that were mostly carried out at country, region, city, and household scales, we explored the influencing factors of the waste generation and reduction at China’s university. One of the top-class universities in China, East China Normal University, was chosen for a case study. Through extending the theoretical framework of planned behavior in the context of waste management, the effect of attitude towards the behavior, perceived behavioral control, and subjective norms, together with personal attributes such as gender, major, grade, and living expenses are investigated quantitatively. Direct weighting and questionnaire survey were conducted in 112 randomly selected dormitory rooms. Totally 523 valid questionnaires were collected. Through descriptive and multivariable regression analyses, the DSW condition, university students’ behavior of waste reduction, and influencing factors are revealed. The results are expected to provide data information for international comparative study, and supplement references for the waste management research in the world. The major findings are concluded as follows.

First, the daily waste generation per student was 0.275 kg, in which residual waste accounted for 64% of total, followed by household food waste (29%), and recyclable waste (7%). The influences of student’s personal attributes, such as financial condition and grade level were significantly and positively correlated with waste output. While the impacts of gender and major varied from waste types. In specific, female students and those from natural sciences produced more residual waste, while male students and those studying social sciences produced more household food waste.

Second, in the theoretical framework of planned behavior, students’ attitude towards waste generation and their perception of the difficulty of waste reduction would significantly affect the generation of waste. The top three ranked difficulties in waste classification and reduction included the time mismatch between waste generation and dumping, unclear of rules and regulations, and far distance from dormitory to dumping sites. The recycle of the idle goods and waste through the flea market was the most preferred way that students were willing to participate in.

Third, the policy suggestions for the university include (a) developing personalized and humanized waste management policies, such as paying attention to female student dormitory for managing the residual waste, while male dormitory for household food waste management, and integrating waste data with GIS model to optimize the site choice of dumping sites. (b) Enhancing student’s environmental awareness through diverse educational activities, such as public and fundamental courses related to waste and resource management, some elective courses combining theories with practices, special lectures, and knowledge competitions. (c) Expanding the publicity role of campus cultural activities on waste reduction, such as building online flea market platform on WeChat, organizing culture and creative products design contest, and holding DIY dormitory waste-oriented product competitions.

## Supplementary Information

Supplementary Information.
