# Supplementary material for: Influencing factors and reduction of domestic solid waste at university dormitory in Shanghai, China
Source: Sci Rep. 2022 Jan 12;12:570. doi: 10.1038/s41598-021-04582-0 (PMC8755736; doi:10.1038/s41598-021-04582-0)
Supplement: Supplementary file 1 — Supplementary Information. [file 41598_2021_4582_MOESM1_ESM.docx]

**Supporting information for**

**Influencing factors and reduction of domestic solid waste at university dormitory in Shanghai, China**

Yuhan Pan^1^, Mengyang Li^2^, Yuanyuan Li^1^, Hongwei Guo^1^, Ji Han^1,3*^

^1^ Shanghai Key Laboratory for Urban Ecological Processes and Eco-Restoration, School of Ecological and Environmental Sciences, East China Normal University, Dongchuan Rd. 500, Shanghai 200241, China

^2^ Logistics Support Department, East China Normal University, Dongchuan Rd. 500, Shanghai 200241, China

^3^ Institute of Eco-Chongming, 3663 N. Zhongshan Rd., Shanghai 200062, China

^*^ Corresponding author E-mail: jhan@re.ecnu.edu.cn

**Appendix A** The questionnaire on the influencing factors of DSW

| **Questions** | **Options** |
| --- | --- |
| What's your dormitory number? |  |
| *you can select only 1 option for the following four questions. | |
| What is your gender? | 🗆male 🗆female |
| What is your grade? | 🗆undergraduate 🗆master 🗆doctor |
| What's your major? | 🗆Philosophy (philosophy, logic, religion, etc.)  🗆Economics (economics, finance)  🗆Law (Marxist theory, sociology, political science, public security)  🗆Pedagogy (pedagogy, physical education)  🗆Literature (Chinese language and Literature, Foreign language and Literature, Journalism)  🗆Art (music, painting, sculpture, animation, dance, etc.)  🗆History (history, archaeology, etc.)  🗆Science (mathematics, physics, chemistry, biology, psychology, electronic information science, statistics, environmental science, etc.)  🗆Engineering (materials, machinery, instrumentation, civil engineering, environmental engineering, chemistry and pharmaceuticals, water conservancy, etc.)  🗆Medicine (Clinical Medicine, Stomatology, Pharmacy, Forensic Medicine, etc.)  🗆Management (Business Administration, Public Administration, Library Archives, etc.)  🗆Agronomy (horticulture, forestry, animal science, aquaculture, etc.) |
| How much is your living expenses per month? | 🗆<500 🗆500-1000 🗆1000-1500 🗆1500-2000 🗆>2000 |
| you can select more than 1 option for the following four questions. | |
| When do you produce waste? | 🗆before 8 a.m. 🗆8-12 a.m. 🗆12-16 p.m.  🗆16-20 p.m. 🗆after 20 p.m. |
| What do you think may cause the increase of waste generation? | 🗆Innovation of shopping such as online shopping  🗆More money available  🗆Follow the roommates' shopping choices  🗆Too much time spent in the dormitories  🗆Increase of basic needs with the development of economy and society |
| What actions you may take to avoid generating more waste? | 🗆Cultivate the financing concept and control the expenditure  🗆Reduce online shopping and go to the physical store more  🗆Practice low-carbon lifestyle and reduce unnecessary expenses  🗆Vitalize the lifestyle and avoid spending too much time in the dormitory |
| What activity would you like to participate in waste reduction and recycling? | 🗆Watch the video promotional documentaries  🗆Attend related lectures  🗆Participate in the old property renovation activities  🗆Sell unused items through the secondary market |
| *Here's an open question, please express your idea freely. | |
| What is the main difficulty in waste classification and reduction? |  |
